# Effect of Diet on the Enteric Microbiome of the Wood-Eating Catfish *Panaque nigrolineatus*

**DOI:** 10.3389/fmicb.2019.02687

**Published:** 2019-11-29

**Authors:** Ryan C. McDonald, Joy E. M. Watts, Harold J. Schreier

**Affiliations:** ^1^Department of Biological Sciences, University of Maryland, Baltimore County, Baltimore, MD, United States; ^2^Department of Biological Sciences, University of Portsmouth, Portsmouth, United Kingdom; ^3^Department of Marine Biotechnology, University of Maryland, Baltimore County, Baltimore, MD, United States

**Keywords:** lignocellulose digestion, microbiome, 16S rRNA gene amplicon sequencing, predictive metagenomics, Amazonian catfish

## Abstract

Wood is consistently found in high levels in the gastrointestinal tract of the Amazonian catfish *Panaque nigrolineatus*, which, depending on environmental conditions, can switch between xylivorous and detritivorous dietary strategies. This is highly unusual among primary wood consumers and provides a unique system to examine the effect of dietary change in a xylivorous system. In this study, microbiome and predictive metagenomic analyses were performed for *P. nigrolineatus* fed either wood alone or a less refractory mixed diet containing wood and plant nutrition. While diet had an impact on enteric bacterial community composition, there was a high degree of interindividual variability. Members of the Proteobacteria and Planctomycetes were ubiquitous and dominated most communities; Bacteroidetes, Fusobacteria, Actinobacteria, and Verrucomicrobia also contributed in a tissue and diet-specific manner. Although predictive metagenomics revealed functional differences between communities, the relative abundance of predicted lignocellulose-active enzymes remained similar across diets. The microbiomes from both diets appeared highly adapted for hemicellulose hydrolysis as the predicted metagenomes contained several classes of hemicellulases and lignin-modifying enzymes. Enteric communities from both diets appeared to lack the necessary cellobiohydrolases for efficient cellulose hydrolysis, suggesting that cellobiose is not the primary source of dietary carbon for the fish. Our findings suggest that the *P. nigrolineatus* gut environment selects for an enteric community based on function, rather than a vertically transferred symbiotic relationship. This functional selection strategy may provide an advantage to an organism that switches between dietary strategies to survive a highly variable environment.

## Introduction

The Amazonian catfish *Panaque nigrolineatus* consumes large quantities of wood as part of its diet, a trait shared among a limited number of related fish species (Schaefer and Stewart, 1993). Depending on environmental conditions, *P. nigrolineatus* can adjust its feeding behavior, switching between xylivorous (“wood-eating”), detritivorous, and herbivorous feeding behaviors. While jaw and tooth morphologies appear to be well adapted for wood consumption (Geerinckx et al., 2007; Adriaens et al., 2008), other features such as GI tract length, microvilli surface area, and gut retention times are inconsistent with a diet dependent primarily on wood consumption (German, 2009; German et al., 2010). It has been hypothesized that wood intake may serve a selective advantage during the Amazonian dry season (Araujo-Lima et al., 1986), or a consequence of epiphyte or fungal hyphae consumption. The nutritional benefits of wood feeding and subsequent digestion by *P. nigrolineatus* are, as yet, unknown.

Consuming wood as a primary food source poses many challenges. The physical and chemical properties of most woods make them highly recalcitrant and of poor nutritive value (Pu et al., 2011). Although rich in carbohydrates, these compounds are largely inaccessible due to their incorporation into structural polysaccharides, such as cellulose and hemicelluloses (Cosgrove, 2005). The crystalline nature of cellulose and presence of other structural plant cell wall polymers, such as lignin, limit microbial infiltration and exclude water, making the environment non-conducive to enzymatic hydrolysis (Cosgrove, 2005; Grabber, 2005). In addition to these challenges, wood is also nitrogen deficient; the nitrogen content of Amazonian woods is regularly below 0.5% (Martius, 1992). To overcome this deficiency, many wood-feeding organisms will also consume non-wood nutrient sources or rely on the activity of enteric microorganisms to supply reduced nitrogen compounds (Raychoudhury et al., 2013; Gruninger et al., 2016; Mikaelyan et al., 2017). With a few exceptions, nutrient acquisition by wood-eaters is mediated by their enteric microbial community, which liberates assimilatory sugars and generates nitrogenous compounds (Tokuda et al., 2014; Cragg et al., 2015).

The enteric bacterial community of *P. nigrolineatus* has been shown to possess a unique microbiome, with the potential of assisting lignocellulose degradation and conducting biological nitrogen fixation (McDonald et al., 2012). Included in these communities are several species of Rhizobiales, Flavobacteriales, Legionellales, Burkholderiales, and Clostridiales. Distinct communities have been identified in the fore, mid, and hindguts of the fish despite any well-defined anatomical features (e.g., sphincters or cecum) demarcating these regions. A diverse and distinct fungal community also resides and is associated with cellulose degradation in the GI tract (Marden et al., 2017). Culture-based analyses and biochemical tests confirmed the presence of a lignocellulolytic and diazotrophic community (Watts et al., 2013; McDonald et al., 2015). These analyses suggest that a considerable amount of microbial metabolic cross-feeding may be occurring within the fish GI tract, where carbohydrates are liberated by cellulolytic species and consumed by non-cellulose utilizers. In comparison to the cellulolytic community, the enteric diazotrophic community was less diverse and was comprised of known nitrogen-fixing Rhizobiales and *Clostridium* (McDonald et al., 2015).

Diet has been shown to have a major impact on enteric microbial communities of animals (David et al., 2014; Ringø et al., 2016). Many xylivores are highly adapted for wood consumption, but retain some capacity to survive on less refractory diets (Tanaka et al., 2006; Miyata et al., 2014). However, the enteric communities are specialized and necessary for the processing of digesta; their manipulation often has deleterious effects on the host (Rosengaus et al., 2011). For some wood-feeding organisms, the enteric communities appear highly stable, where dietary changes have minimal impact on community composition (Boucias et al., 2013; Wang et al., 2016). Dual feeding behavior in adult fish is not unique to *P. nigrolineatus*, but far less common to the ontogenetic diet shifts seen in other fish species (Bledsoe et al., 2016; Sánchez-Hernández et al., 2019). To examine the effects of diet on a primarily wood-feeding fish species, we characterized the enteric microbiomes of animals fed either a wood or mixed diet.

## Materials and Methods

### Animals, Experimental Design, and Tissue Sampling

*Panaque nigrolineatus* (L190) were acquired through aquarium wholesalers. Fishes were randomly assigned to aerated, filtered tanks (29 ± 1°C), and acclimated for 3 weeks on an *ad libitum* mixed diet of red palm (*Cocos nucifera*), hearts of palm (*Euterpe precatoria*), and sinking algae wafers (Hikari, Hayward, CA). After acclimation, fishes were randomly assigned to either a mixed or wood diet for 6 weeks. Mixed diet fishes were provided algae wafers and hearts of palm daily with continuous access to wood. Wood-fed fishes were provided with wood exclusively. All wood was autoclaved twice prior to being placed in the tanks. To inhibit algae growth, all fishes were reared under low/no light conditions. Lights were turned on approximately 30 min each day for tank maintenance and feeding. Two independent feeding studies were carried out, designated feeding study 1 (two fishes, designated 1X and 1W, for mixed (X)-and wood (W)-fed diets, respectively) and feeding study 2 (six fishes, designated 2X, 2W, 3X, 3W, 4X, and 4W). Both feeding studies were conducted under identical experimental conditions.

At the conclusion of the feeding experiments, fishes were euthanized *via* an overdose of the anesthetic 3-aminobenzoic acid ethyl ester (MS-222, 50 mg/L) and were immediately transferred to a chilled dissecting tray where the ventral body plate was removed. The body cavity was filled with cold, sterile phosphate buffered saline (PBS) to facilitate removal of the GI tract. Once uncoiled, the intestines were disconnected by cutting at the anus and just posterior to the stomach, and measured for length. The intestine was divided into three equal lengths demarcating the fore (F), mid (M), and hindgut (H).

### DNA Extraction, Amplification, and Sequencing

GI tract samples were processed for fish from each diet using the Qiagen (Germantown, MD, USA) DNeasy Blood and Tissue Kit with pre-treatments for Gram-positive and Gram-negative bacteria according to the manufacturer’s instructions. DNA was extracted from three samples of each GI tract region for each fish and was pooled and processed for PCR amplification. To profile the bacterial community, 16S rRNA gene sequencing libraries were prepared according to the manufacturer’s instructions (Illumina, San Diego, CA). Briefly, the V3-V4 region of the 16S rRNA gene was amplified using the primer pair evaluated previously (Klindworth et al., 2017) using the 2X KAPA HiFi HotStart ReadyMix (Sigma-Aldrich, St. Louis, MO) with the following polymerase chain reaction (PCR) program parameters: an initial denaturation step of 3 min at 95°C followed by 25 cycles of denaturation for 30 s at 95°C, annealing for 30 s at 55°C, and elongation for 30 s at 72°C, followed by a final elongation for 5 min at 72°C. Index PCR was performed using the Nextera XT Index Kit according to the manufacturer’s instructions (Illumina, San Diego, CA). PCR products purified using AMPure XP beads (Beckman Coulter, Brea, CA), pooled in equimolar amounts, and sequenced using the Illumina MiSeq platform (250 bp paired-end reads). For the second feeding study, the standard Illumina primers for V3-V4 were modified to include a unique trinucleotide sequence (Table 1) between the overhang adapter and the 16S rRNA primer, which allowed for double-dual indexing of the samples (Fadrosh et al., 2014). A total of 10 samples were pooled in equimolar amounts prior to the standard index PCR reaction.

**Table 1:** List of modified Illumina V3-V4 primers and resulting read counts from the second feeding study 16S rRNA gene survey.

	Sample ID	Forward primer	Reverse primer	PCR amplification	Demultiplexed read count
Pooled sample 1	Wood1	F1	R1	−	−
	Wood2	F2	R1	−	−
	XTWa	F3	R1	+	58,488
	WTW	F1	R2	+	81,646
	2WF	F2	R2	−	−
	2WM	F3	R2	+	44,379
	2WH	F1	R3	+	40,820
	3WF	F2	R3	−	−
	3WM	F3	R3	+	44,457
	3WH	F1	R4	+	25,011
Pooled sample 2	2XF	F1	R1	+	7,298
	2XM	F2	R1	+	12,536
	2XH	F3	R1	+	22,560
	3XF	F1	R2	+	5,149
	3XM	F2	R2	+	14,524
	3XH	F3	R2	+	28,446
	4XF	F1	R3	−	−
	4XM	F2	R3	+	12,715
	4XH	F3	R3	+	10,219
	XTWb	F1	R4	+	43,047
Forward primer structure		**5′-Overhang_adapter – Trimer_Index – Forward_16S_primer-3′**			
	F1	5′-TCGTCGGCAGCGTCAGATGTGTATAAGAGACAG-GTT-CCTACGGGNGGCWGCAG			
	F2	5′-TCGTCGGCAGCGTCAGATGTGTATAAGAGACAG-CAT-CCTACGGGNGGCWGCAG			
	F3	5′-TCGTCGGCAGCGTCAGATGTGTATAAGAGACAG-TCC-CCTACGGGNGGCWGCAG			
Reverse primer structure		**5′-Overhang_adapter - Trimer_Index – Reverse_16S_primer-3′**			
	R1	5′-GTCTCGTGGGCTCGGAGATGTGTATAAGAGACAG-CCA-GACTACHVGGGTATCTAATCC			
	R2	5′-GTCTCGTGGGCTCGGAGATGTGTATAAGAGACAG-TGA-GACTACHVGGGTATCTAATCC			
	R3	5′-GTCTCGTGGGCTCGGAGATGTGTATAAGAGACAG-GTA-GACTACHVGGGTATCTAATCC			
	R4	5′-GTCTCGTGGGCTCGGAGATGTGTATAAGAGACAG-TAC-GACTACHVGGGTATCTAATCC			

*The primers were modified to include a unique trinucleotide sequence between the 16S rRNA primer and overhang adapter. Successful amplification from the sample is indicated by (+)*.

### DNA Sequence Processing and Community Analysis

Raw reads were preprocessed using CLC Workbench (version 9) (Qiagen). Adapter sequences were removed and read pairs were quality trimmed (qual. limit = 0.05; ambiguous nucleotide maximum = 2; minimum sequence length = 100 bp) and merged (mismatch cost = 2; gap cost = 3; maximum unaligned = 0; minimum score = 8). Sequences were analyzed using the Quantitative Insights Into Microbial Ecology (QIIME) bioinformatics pipeline (Caporaso et al., 2010). Operational taxonomic units (OTUs) were picked using the open reference method against the Silva_132 database (minimum OTU cluster size = 2; OTU similarity = 0.97) (Desantis et al., 2006). Taxonomies were summarized at multiple levels (L2-L6) using the *summarize_taxa.py* script. Rarefaction plots and alpha diversity measures were calculated using the *alpha_rarefaction.py* and *alpha_diversity.py* scripts, respectively. OTU matrices were normalized using DESeq2 variance stabilizing transformation prior to Bray-Curtis distance matrix generation. Principle coordinate analysis (PCoA) plots were generated using *principal_cordinates.py*. OTU networks were generated for all tissue samples using the *make_otu_network.py* script. For this analysis, OTUs were re-picked according to the above method; however, the minimum cluster size was increased to 25 in order to reduce the number of nodes. The network was visualized using Cytoscape v3.2.1 using an edge-weighted spring-embedded layout.

### Predictive Functional Profiling of Microbial Communities

Predictive functional profiling was performed using Phylogenetic Investigation of Communities by Reconstruction of Unobserved States (PICRUSt) (Langille et al., 2013). For the PICRUSt analysis, all samples were normalized to 80,000 reads/sample prior to OTU picking. OTUs were picked using the closed reference method against gg_13_8 (minimum OTU cluster size = 2; OTU similarity = 0.94). The resulting BIOM table was normalized for 16S rRNA gene copy number prior to predicting functions for metagenomes. For this analysis, KEGG orthologs (KO) were recorded. KOs were collapsed into pathways (L1-L3) using the *categorize_by_function.py* script. The results were visualized in Rstudio (v 0.98.1083) using the heatmap.2 function of the gplots package. To determine which OTUs and samples were contributing particular functions, *metagenome_contributions.py* was also run. To determine if functional profiles differed across tissue region or diet, principal component analysis (PCA) was performed on the predicted metagenome (not collapsed within the KEGG hierarchy) using the ggplot2 package in Rstudio.

## Results

### Microbial Community Characterization

Microbial communities were analyzed from GI tract regions of wood-fed and mixed diet-fed fish by next generation 16S rRNA amplicon sequencing, and functional gene profiles were then extrapolated using *in silico* methods. Except for foregut samples from two wood-grown and one mixed-diet fish, which could not be amplified by PCR, more than 1.2 × 10^6^ high quality reads were generated across all samples and redistributions are shown in Table 1. Rarefaction analysis (Supplementary Figure S1) and alpha diversity measures (Table 2) showed that the bacterial communities were sufficiently sampled, and further sequencing would be unlikely to significantly increase the observed microbial diversity detected. Species richness varied greatly between feeding study replicates, with a marked reduction in diversity observed in the second feeding experiment. Despite this, Good’s coverage estimates remained high (>0.95 for all samples).

**Table 2:** Observed OTUs, Chao_1_, and Good’s coverage values were calculated to compare bacterial diversity among diets and tissue regions.

Sample name	Diet	GI tract region	Feeding study group	Observed OTUs	Chao_1_	Good’s coverage
1XF	Mixed	Foregut	1	2,418	3,051	0.989
2XF		Foregut	2	465	550	0.979
3XF		Foregut	2	520	673	0.957
1XM		Midgut	1	2,246	3,079	0.984
2XM		Midgut	2	637	751	0.982
3XM		Midgut	2	577	663	0.987
4XM		Midgut	2	601	662	0.989
1XH		Hindgut	1	2,355	3,131	0.988
2XH		Hindgut	2	517	624	0.992
3XH		Hindgut	2	437	516	0.994
4XH		Hindgut	2	699	813	0.979
XTWa		Tank water	2	1,517	1,586	0.995
XTWb		Tank water	2	1,463	1,551	0.993
1WF	Wood	Foregut	1	962	1,145	0.994
1WM		Midgut	1	2062	2,161	0.993
2WM		Midgut	2	1,232	1,481	0.990
3WM		Midgut	2	1,191	1,401	0.991
1WH		Hindgut	1	3,021	3,136	0.991
2WH		Hindgut	2	901	1,108	0.992
3WH		Hindgut	2	683	820	0.990
WTW		Tank water	2	2,515	2,598	0.995

*All calculations were performed using QIIME with 0.97 OTU identity threshold*.

### Effect of Diet on Enteric Microbial Community Composition

**Figure 1:**
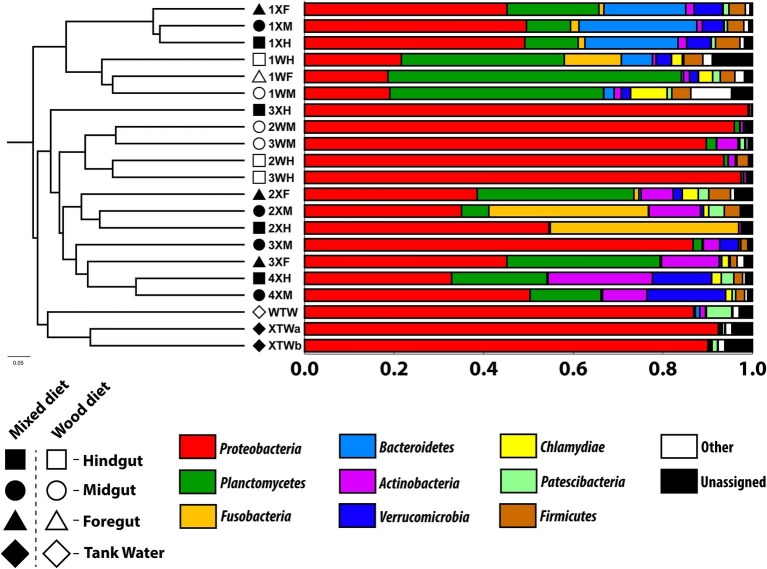
Unweighted pair group method with arithmetic mean (UPGMA) tree based on Bray-Curtis distance analysis. Bar graph represents the relative abundance of predominant bacterial phyla in the tank water (TW, diamond) as well as fore (F, triangle), mid (M, circle), and hindguts (H, square) of *P. nigrolineatus* fed either a wood (W, open symbols) or mixed diet (X, closed symbols). OTUs were picked using the SILVA_132 16S database (OTU ID =0.97).

All microbial communities analyzed were dominated by a few microbial phyla (Figure 1). These included Proteobacteria (19–99%), Planctomycetes (<1–66%), Fusobacteria (0–43%), Bacteroidetes (<1–26%), and Actinobacteria (<1–23%). While the relative abundance of these major phyla varied greatly across diet, tissue region, and feeding study replicate, the constituent members of these phyla largely remained unchanged (Supplementary Table S1). Planctomycetes were observed in high abundance in all the first experimental study samples, as well as the foreguts of mixed diet fish from the second feeding study; the most predominant classes were Pirellulales, Gemmatales, Planctomycetales, and Isosphaerales. Sequences with the highest sequence similarity with an unidentified *Cetobacterium* species in the class Fusobacteria were observed in the mid and hindgut of a single mixed (X) diet fish (2XM and 2XH) as well as the hindgut of a wood-fed (W) fish (1WH).

In the first experiment, Bacteroidetes were predominant in all tissue regions of the mixed diet fish, as well as the hindgut of the wood-fed fish. While the diversity of Bacteroidetes was high, most sequences had high sequence similarity to the endosymbiont *Candidatus* Cardinium. However, in both experiments, Actinobacteria was the most common phyla, found in nearly all tissue regions across feeding regimens and feeding study replicates. The highest proportions of Actinobacteria were identified in the mixed diet fish of the second feeding experiment and had high sequence similarity to the Corynebacteriales genera *Mycobacterium* and *Gordonia*.

Both the Proteobacteria and Verrucomicrobia populations showed marked composition differences between the two feeding study replicates (Figure 1). The Proteobacteria identified in the first experiment were predominantly Alphaproteobacteria, consisting of Rhizobiales and Rhodobacterales. The Proteobacteria from the remaining enteric samples of the second experiment were almost exclusively Gammaproteobacteria and consisted of Aeromonadales. Distinct differences in Proteobacteria were also identified in the tank water; sequences most closely aligned with Rhodospirillales were foremost in mixed diet tank water, while Sphingomonadales-like sequences dominated wood diet tank water. Verrucomicrobia was identified predominantly in the mixed diet-fed fish of both feeding studies. In the first experiment, the sequences had highest similarity to an uncultivated Verrucomicrobiales and *Luteolibacter* species, contrasting to the second experiment where the sequences were most similar to either a species of *Chthoniobacter* or *Prosthecobacter*.

**Figure 2:**
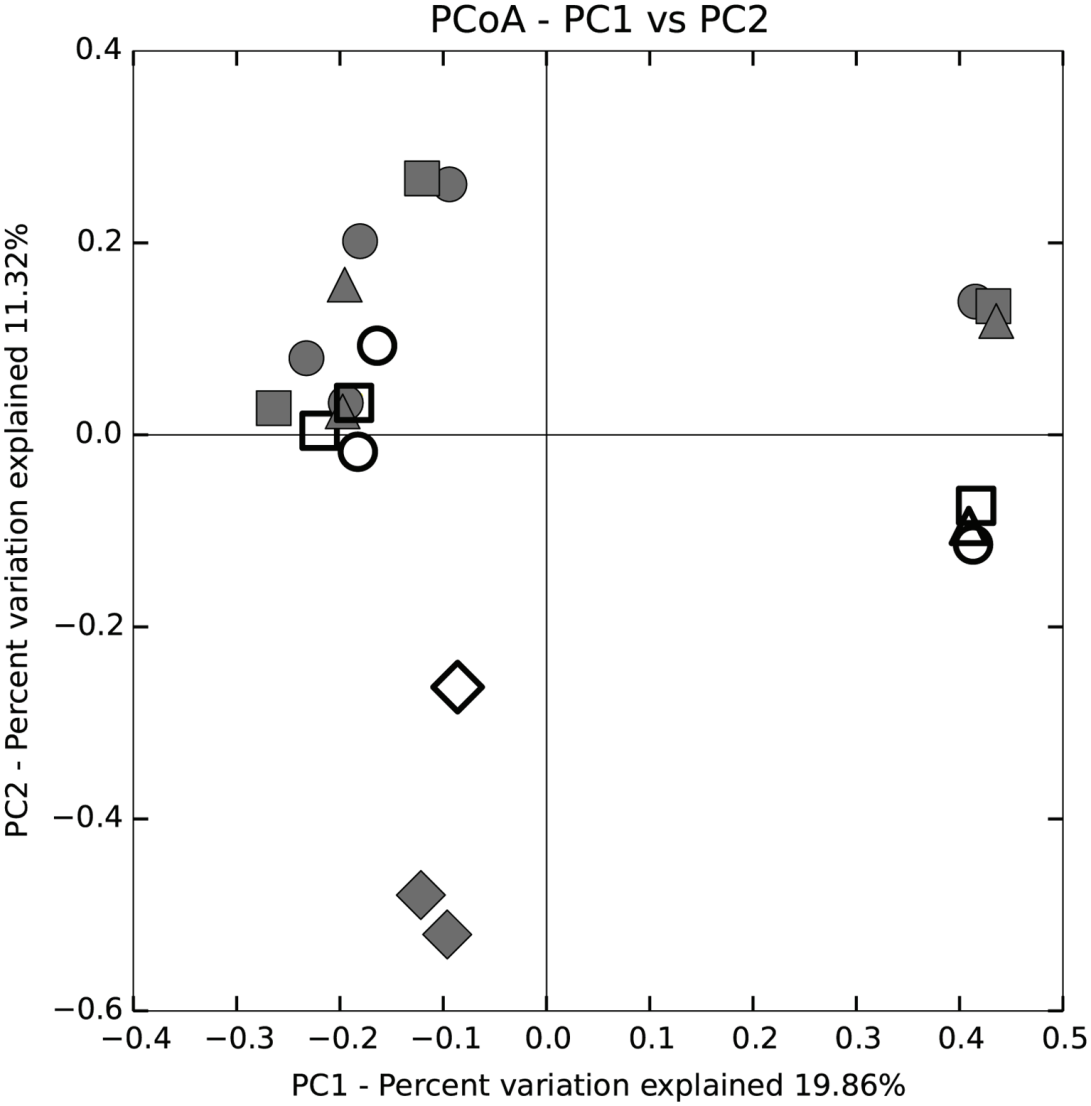
Principal coordinate analysis (PCoA) based on Bray-Curtis distance analysis of fore, mid, and hindgut and enteric and tank water microbial communities of *P. nigrolineatus* fed either a wood or mixed diet. Diets and GI tract region identifiers are indicated in the legend to Figure 1.

Trends in community composition were detected; however, diet did not appear to select for specific microbial communities in any tissue region of the *P. nigrolineatus* GI tract. PCoA and UPGMA trees based on Bray-Curtis distances showed samples largely clustered based on experimental study and diet (Figures 1, 2). Feeding experiment 1 and 2 were distinguished along PC1 of the PCoA. UPGMA trees revealed that within experimental study groups, wood and mixed diet samples largely formed monophyletic clades. The exception to this is sample 3XH, which formed a paraphyletic group with the other mixed diet samples from the second experimental feeding group.

*P. nigrolineatus* possesses a taxonomically restricted core microbiome. Fifty-five OTUs were identified in >70% of samples across both feeding regimens (Supplementary Table S2). The majority of these OTUs had high sequence similarity to species of Pirellulales, Rhizobiales, Rhodobacterales, and Aeromonadales, and their relative abundance was highly variable. For this analysis, OTU relative abundance was not used to define the core microbiome (e.g., only including OTUs that represent >1% of all reads). Distinct core microbiomes were observed between wood and mixed diet-fed fish (Supplementary Tables S3, S4). Wood-fed fish had an expanded core microbiome (115 OTUs) relative to mixed diet-fed fish (72 OTUs) (Supplementary Figure S2), with minimal overlap between the two groups (~21%) (Supplementary Figure S3). Despite, minimal OTU overlap, the core microbiomes were taxonomically compositionally similar (Supplementary Tables S2, S3).

**Figure 3:**
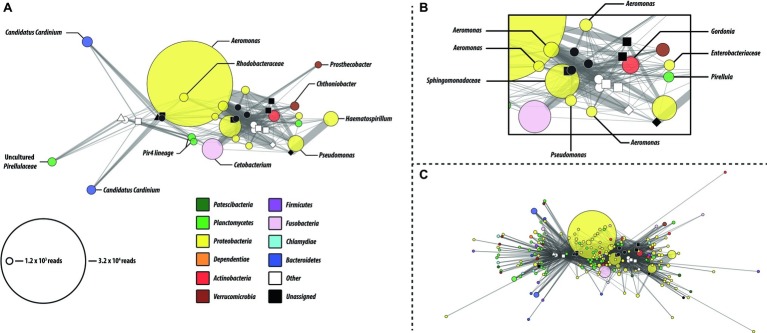
OTU network analysis of microbial communities identified in the GI tract fore, mid, and hindgut, and tank water of *P. nigrolineatus* fed either a wood (open symbols) or mixed diet (closed symbols). **(A)** Reduced OTU network depicting only the most prevalent OTUs (minimum cluster size =1.2 × 10^3^). Node color and size denote consensus taxonomy (phylum) and OTU size, respectively. Edges connecting OTU and sample indicate the presence of the OTU in that particular sample. For instance, where an OTU is present in multiple samples, edge weights indicate the relative number of reads provided by that sample to the OTU. **(B)** Subset of shared OTU nodes from the reduced OTU network. **(C)** Complete network containing all OTUs (minimal cluster size =100). Sample node identifiers are indicated in the legend of Figure 1.

Network analysis revealed that a small number of predominant core microbiome OTUs with high sequence similarity to members of the genera *Aeromonas* were primarily responsible for the observed shift in Proteobacteria abundance (Figures 3B,C). Most OTUs were shared among multiple gut regions, within and across feeding regimens. The reduced OTU network (Figure 3A) represents the most abundant OTUs (minimum OTU cluster size of 1 × 10^3^ reads after rarefaction) and are identical with those identified in the core microbiome analysis. Consistent with the PCoA analysis, more OTUs were shared among samples within a feeding study. Sample node degree distributions also suggest increased biodiversity within the first feeding study (Supplementary Figure S4). This is consistent with the alpha diversity measures. Because the network analysis is constructed from a rarefied OTU table, the reduction in average node degree distributions likely represents a true reduction in biodiversity in these samples and is not a result of reduced sampling depth.

### Predictive Metagenome Profiling

**Figure 4:**
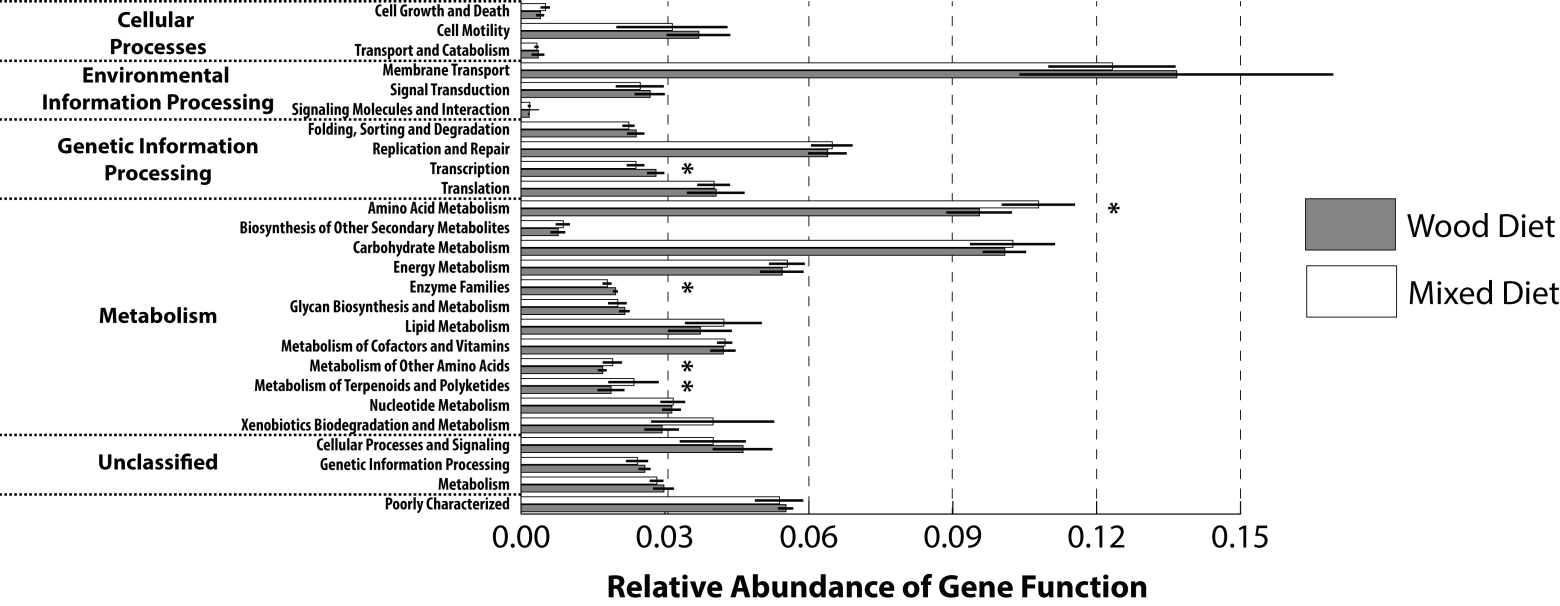
Predictive functional profiles generated from 16S rRNA marker gene sequences using PICRUSt. Functional profiles were generated based on KEGG ortholog prediction and collapsed into higher pathways (L2), according to the KEGG pathway database. KEGG orthologs assigned to human disease and organismal systems pathways were excluded from the analysis. Significant differences between tank water and GI tract regions from wood and mixed diet-fed fish were observed for several cellular processes (two-tailed Student’s *t*-test; ^*^p > 0.05). Diets and GI tract region identifiers are indicated in the legend to Figure 1.

To gain insight into the metabolic capacity of the enteric microbiome, a PICRUSt analysis was performed to generate a predictive functional profile. To compare across feeding regimens, the predicted gene profiles from the mid and hindguts were averaged and collapsed at a higher level (L2) within the KEGG hierarchy (Figure 4). Based on this analysis, the relative abundance of several pathways was statistically different between feeding regimens. Wood-fed fish had a higher abundance of genes involved in transcription and enzyme families that include peptidases, cytochrome P450, and protein kinases, while mixed diet fishes were enriched for genes involved in the metabolism of amino acids, terpenoids, and polyketides, as well as the metabolism of other amino acids. These differences were reflected in the PCA analysis of the predicted microbiome, which shows clustering of wood and mixed diet samples along PC2 (Supplementary Figure S5). No significant differences were observed for the relative abundance of genes involved in carbohydrate metabolism or xenobiotic degradation. There were no significant differences in pathway relative abundances between the mid and hindgut regions within feeding regimens suggesting the microbiome within these tissues may function similarly in regard to lignocellulose degradation. This finding was supported by PCA analysis (Supplementary Figure S6), which showed no distinction between any tissue regions.

Despite high similarity between wood and mixed diet-fed fish predicted metagenome at higher levels in the KEGG hierarchy, comparisons at lower levels (L3) revealed many differences (Supplementary Table S5). The majority of these KEGG pathways were associated with the metabolism of amino acids and carbohydrates. Mixed diet fish had an increase in pathways associated with amino acid transformation and included the metabolism of glycine, serine, threonine, histidine, lysine, tryptophan, and tyrosine, as well as the degradation of lysine valine, leucine, and isoleucine. However, pathways associated with carbohydrate utilization were found in high abundance in both feeding regimens. Mixed-diet fish had a higher relative abundance of pathways involved in the metabolism of butanoate, glyoxylate, dicarboxylate, propanoate, and pyruvate. In comparison, wood-fed fish had higher abundance of pathways involved with metabolism of amino and nucleotide sugars, fructose, mannose, galactose, starch, and sucrose, as well as the interconversion of pentose and glucuronate and a higher abundance of genes associated with degradation of other glycans. Additionally, pathways were found to be differentially abundant between feeding regimens and included processes involved with the metabolism of lipids, cofactors, and vitamins, as well as the degradation of xenobiotics.

**Figure 5:**
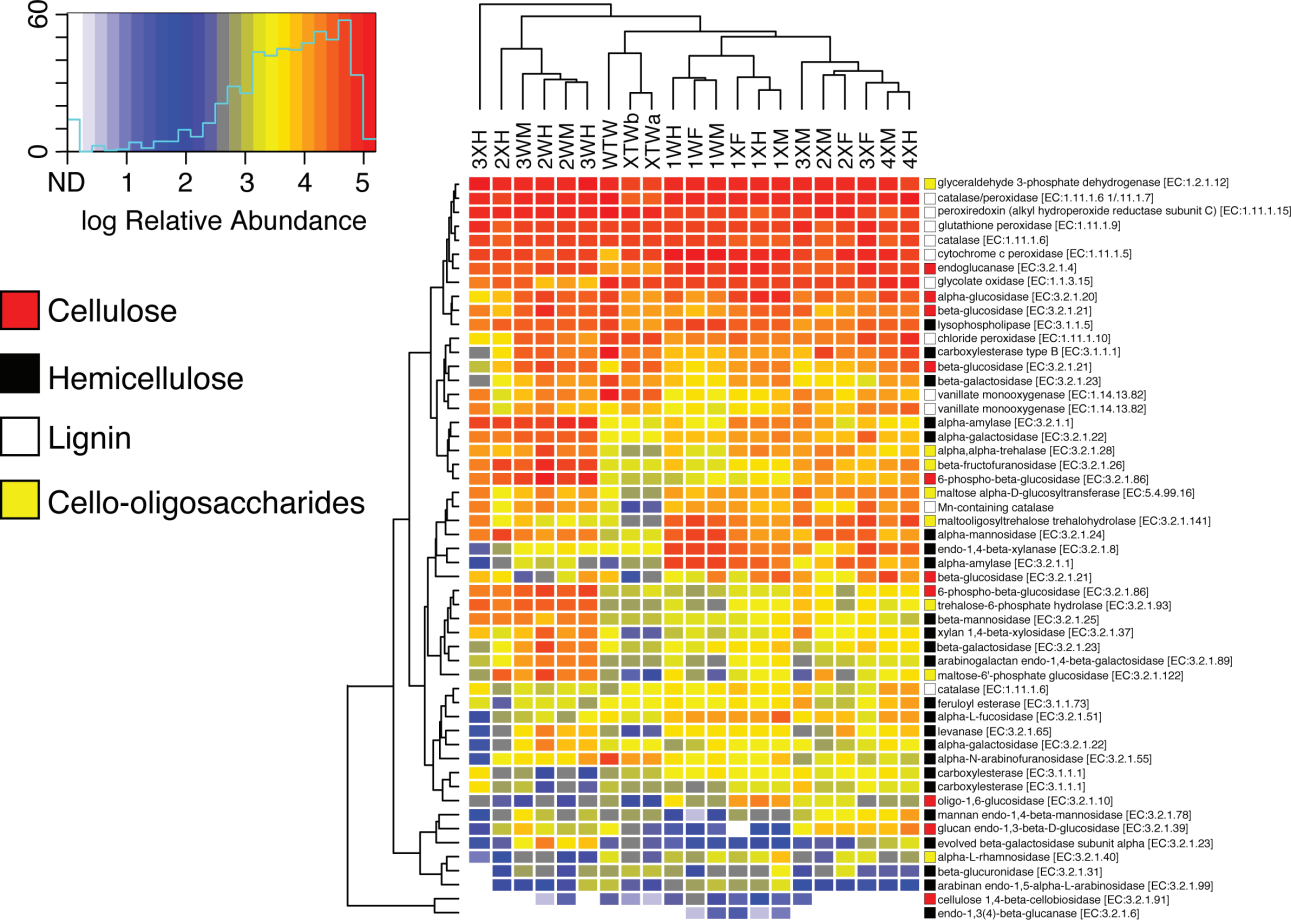
Relative abundance of lignocellulose-active enzymes across diet and tissue type. OTUs were picked against the Greengenes 13_8 16S rRNA database. Predictive metagenomics was performed using Tax4Fun, and heatmaps were generated in R using the heatmap.2 function of the gplots package. Cell color indicates relative abundance among all predicted genes. Colored squares to the right of the heatmap correspond with type of substrate the enzyme is active against. For instances of duplicated E.C. numbers, each row represents a different entry in the KEGG database, each with a unique K-number.

Because wood degradation requires a diverse suite of enzymes for complete hydrolysis, the relative abundance of genes for several lignocellulose-active enzymes were also examined (Figure 5). Included in the analysis were enzymes active against cellulose, hemicellulose, lignin, and cello-oligosaccharides. Despite statistically significant differences in starch metabolism pathways at lower KEGG classifications, there were very few differences in the relative abundance of lignocellulose active enzymes. For most enzymes, neither diet nor tissue region appeared to influence relative abundances with the only significant differences between wood and mixed diet-fed fish seen in the relative abundances of lysophospholipase (EC 3.1.1.5) and carboxylesterase (EC 3.1.1.1), which both act upon hemicellulose. The vast majority of carbohydrate active enzyme genes were observed infrequently (~1 × 10^−6^ to 1 × 10^−5^); however, a limited number were predicted to be in higher abundance (>5.0 × 10^−4^). These were primarily limited to activities likely associated with lignin degradation (e.g., cytochrome C peroxidase, catalase-peroxidase, and glycolate oxidase), but also included glyceraldehyde 3-phosphate dehydrogenase.

**Figure 6:**
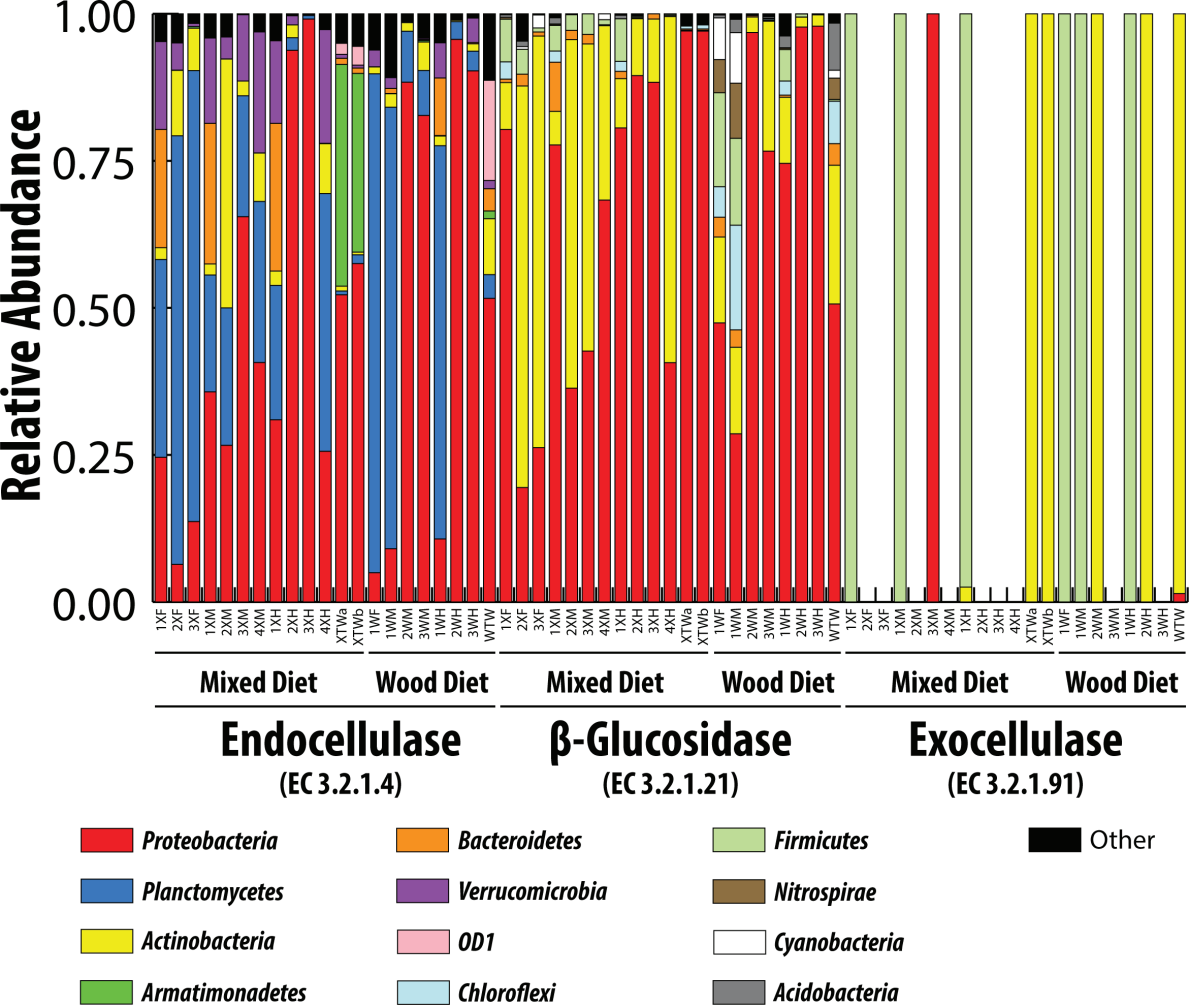
Taxonomic contributions to three classes of cellulases by different bacterial phyla. Classifications were based on consensus taxonomy resulting from the PICRUSt analysis.

Endocellulases (EC 3.2.1.4), exocellulases (EC 3.2.1.91), and ß-glucosidases (EC 3.2.1.21) were detected in both feeding regimens (Figure 5 and Table 3). The relative abundance of the individual cellulases varied (~1 × 10^−7^ to 4 × 10^−4^) but remained largely the same across tissue regions and diets. All tissue regions across both diets had a higher relative abundance of endoglucanases and ß-glucosidases than exocellulases (Table 3). Exocellulases were exceedingly rare in all samples and completely absent from several samples of the second feeding study. Distributions were calculated for each of the three cellulases in the predicted metagenome (Figure 6) and, like the relative abundance analysis, differences were observed between various classes of cellulases. Endocellulases were primarily attributed to Proteobacteria and Planctomycetes in both feeding regimens, while Bacteroidetes, Verrucomicrobia, Armatimonadetes, and Actinobacteria provided functions in specific samples. ß-Glucosidases were provided primarily by Proteobacteria and Actinobacteria. While the relative abundance of Actinobacteria and Proteobacteria varied between samples and diets, the taxonomic makeup was highly consistent and included the Proteobacteria genera *Enterobacter*, *Aeromonas*, *Citrobacter*, *Novospirillium*, *Cronobacter*, and *Rhodobacter*, as well as the Actinobacteria genus *Gordonia* and an unidentified Microbacteriacea. Exocellulases were provided by a single phylum of bacteria in nearly all enteric samples. In the first study, exocellulases were found to be exclusively represented by the Firmicutes and included members of the orders OPB54 and Clostridiales, while in the second study, they originated either Proteobacteria or Actinobacteria and included Rhizobiales and Actinomycetales.

**Table 3:** Relative abundance of the three classes of cellulose degrading enzymes based on predictive metagenomics.

	Mixed diet	Wood diet
Endoglucanase (EC:3.2.1.4)	4.01 × 10^−4^ ± 1.43 × 10^−4^	4.50 × 10^−4^ ± 8.74 × 10^−5^
β-Glucosidase (EC:3.2.1.21)	1.25 × 10^−4^ ± 9.21 × 10^−5^	2.59 × 10^−4^ ± 1.90 × 10^−4^
Cellulose 1,4-β-cellobiosidase (EC:3.2.1.91)	1.04 × 10^−7^ ± 2.03 × 10^−7^	6.19 × 10^−7^ ± 8.98 × 10^−7^

*Abundances were calculated using PICRUSt (see methods) and compared across diet type and tissue region*.

## Discussion

Diet has been shown to have a major impact on enteric bacterial communities of a variety of animals. *P. nigrolineatus* provides a unique opportunity to examine the effects of diet change on a wood-consuming organism. Unlike other xylivorous animals, *P. nigrolineatus* is capable of shifting between diets with seemingly minimal deleterious health effects. In this study, enteric microbial communities were examined for *P. nigrolineatus* provided either a wood or mixed diet. Attention was given to abundance and distribution of putative lignocellulolytic microorganisms that are essential for deriving nutrition from a wood diet. Predictive metagenomics were also used to determine whether a wood diet enriched for pathways involved in lignocellulose degradation.

### Enteric Bacterial Community Composition

Difficulties in obtaining large numbers of fish at one time necessitated two feeding experiments. Any differences between community composition for the two feeding studies might be attributed to the environment where the animals were caught and their initial handling. While our study would have benefitted from examining all animals at the same time, we found that enteric bacterial community composition for both feeding studies were largely consistent with those previously established by previous 16S rRNA metagenomic library analyses (McDonald et al., 2012). Therefore, we believe that communities associated with either mixed diet or wood-fed fish are representative of their dietary regimen and differences observed for community structure are significant and diet influences the *P. nigrolineatus* enteric bacterial community. These results diverge from studies in termites where non-wood diets had minimal impact on bacterial community composition but did cause changes in relative bacterial abundance (Huang et al., 2013; Wang et al., 2016; Su et al., 2017). There was no obvious correlation between diet and OTU richness in any tissue region. Wood-fed fish in the first feeding experiment had a less diverse enteric community than mixed diet-fed fish, a trend that was reversed in the second feeding experiment. These results were inconsistent with studies showing that fishes that are dietary specialists have higher microbial diversity than dietary generalists (Bolnick et al., 2014).

Fish from the first feeding experiment had a higher relative abundance of Planctomycetes (~10–66%) than fish from the second feeding study (<1–35%). Most Planctomycetes were members of the class Pirellulales but also included Gemmatales, Isosphaerales, and Planctomycetales. Planctomycetes have been identified in variety of environments including the GI tracts of fish and termites, soils, and bioreactors (Van Kessel et al., 2011; Wang et al., 2011; Abdul Rahman et al., 2015). Although some Planctomycetes appear to be responsible for heteropolysaccharide degradation in diverse environments, this activity has not been described in Pirellulaceae (Wilhelm et al., 2019).

Proteobacteria were ubiquitous in all samples and were represented by several classes including alpha, beta, gamma, and delta. A higher proportion was observed in the second feeding experiment (~33–99%) than the first (~19–50%). Several proteobacterial families were distributed in a feeding study-, tissue-, and diet-specific manner. In the first feeding study, these families consisted primarily of Rhizobiales, Rhodobacterales, and Aeromonadales, while those from the second feeding study consisted of Rhodobacterales, Rhodospirillales, Aeromonadales, Betaproteobacteriales, Enterobacteriales, Legionellales, and Pseudomonadales. The diversity of Proteobacteria in mixed diet-fed fish of the second feeding study was higher than the wood-fed fish community, which was dominated by Aeromonas and Enterobacterales. Many of these families of Proteobacteria as well as Planctomycetes have been associated with fasting fish, but their role in the wood-fed fish is unclear (Kohl et al., 2014; Xia et al., 2014). Aeromonas has been identified as a major contributor of cellulases in the GI tract of herbivorous grass carp *Ctenopharyngodon idella*, but is also a known pathogen of fresh water fishes. Its role in *P. nigrolineatus* is unclear (Jiang et al., 2011; Ran et al., 2018).

Several bacterial phyla were highly represented in limited number of samples from both feeding studies. These included Actinobacteria, Fusobacteria, Bacteroidetes, and Verrucomicrobia. Actinobacteria were predominantly identified in the second feeding study mixed diet fish and were comprised of species of *Gordonia* and *Mycobacterium*. Both genera have been identified as major components of fish microbiomes where they may play a role in xenobiotic and cellulose degradation and enhance the growth rates of the host (Arenskötter et al., 2004; Medie et al., 2010; Sheikhzadeh et al., 2017). The Bacteroidetes had high sequence similarity to *Candidatus* Cardinium, which are known obligate intracellular pathogens of arthropods and can regulate host health, development, and reproduction (Giorgini et al., 2009). Organisms related to *Candidatus* Cardinium have been identified in plant pathogenic nematodes (Noel et al., 2006) as well as intracellular symbionts of several plant-feeding arthropods (Zchori-Fein and Perlman, 2004; Zhang et al., 2013). Fusobacteria sequences had high sequence similarity to species of *Cetobacterium*. Members of this genus have been identified in high abundance in the GI tracts of a variety of fish species where they may play a role in vitamin synthesis (Liu et al., 2016; Ramírez et al., 2018). Verrucomicrobia were identified as members of the genera *Prosthecobacter* and *Chthoniobacter* in the first and second feeding studies, respectively. They are often plant-associated and exist as endophytes or members of the rhizospheric community (Rascovan et al., 2016; Dong et al., 2018).

### Predicted Metagenome Reconstruction

Any changes in diet that may have influenced the metabolic capacity of the enteric microbial community was examined by predictive metagenomics, which revealed functional differences at higher KEGG classifications. Relative to the mixed diet, the microbiomes of the wood-fed *P. nigrolineatus* did not appear to be enriched for genes involved in lignocellulose degradation. Several studies have demonstrated the ability of microbial communities to shift in response to changes in resource availability (Muegge et al., 2011; Gomez et al., 2016). The relative lack of a reduced metabolic response in *P. nigrolineatus* suggests either top down regulation by the host, i.e., the GI tract environment selects for microbes with specific functional capacities independent of diet, or that the mixed and wood-only diets were not different enough to drive divergence in gut microbiome function. However, studies have shown gut environments to select for specific microbial functions independent of the taxonomy of the microorganisms (Lozupone et al., 2008; Sanders et al., 2015).

Diet had minimal impact on the relative abundance of microbial carbohydrate-active enzymes. Of the 36 investigated genes involved in cellulose, hemicellulose, and cello-oligosaccharide metabolism, a single gene, encoding lysophospholipase (E.C. 3.1.1.5), was significantly more abundant in the wood-fed fish. Conversely, a single gene, encoding carboxylesterase (E.C. 3.1.1.1), was predicted to be in higher abundance in mixed diet-fed fish. Despite this, the microbiomes of both diets appeared to be adapted to metabolize plant polysaccharides. The relative abundance of the three major cellulases was similar to those reported for nitrogen amended green plant waste bioreactors using the same PICRUSt method (Yu et al., 2017). Consistent with our findings, the study also reported large variations in the relative abundances of the different cellulases, with endocellulases and β-glucosidases estimated to be much more abundant (~100×) than exocellulases. The relative proportion of β-glucosidases to cellulases in bacterial genomes are generally lineage specific (Berlemont and Martiny, 2013), but there are typically fewer exocellulases than endocellulases and β-glucosidases in the genomes of true cellulose utilizers. However, these genes are not known to exist at ratios approaching 1:100, which we have identified here. Similar ratios of cellulases have been found in the predicted metagenomes of other environmental and enteric microbiomes known to hydrolyze plant polysaccharides (Zheng et al., 2018; Gao et al., 2019). This suggests either a limitation of PICRUSt to accurately predict the abundance of one or more classes of cellulases. Or, alternatively, the skewed ratios are indicative of a large population of microbial opportunists who do not hydrolyze cellulose directly, but are capable of exploiting the disaccharides and short oligosaccharides released by cellulolytic species.

The presence of a large, opportunistic cellulose-utilizing community was confirmed by analysis of cellulase contributions. Distinct taxonomic lineages contributed each of three major cellulases. Endocellulases were contributed primarily by Planctomycetes, Proteobacteria, Bacteroidetes, Actinobacteria, and Verrucomicrobia, while β-glucosidases were provided by Proteobacteria and Actinobacteria. This contrasts sharply to exocellulases, which were contributed almost exclusively by either Firmicutes or Actinobacteria in the first or second feeding studies, respectively. Many of the bacterial orders identified as contributing the endocellulases and β-glucosidases were also identified as major constituents of the microbial community. These included Pirellulales, Gemmatales, and Planctomycetales of the Planctomycetes as well as Rhodobacterales, Rhizobiales, Aeromonadales, Enterobacterales, Pseudomonadales, Verrucomicrobiales, and Cytophagales of the Proteobacteria. Endocellulases and β-glucosidases are common among non-cellulose utilizers. While there are true lignocellulose utilizing members of these orders, the majority utilize these enzymes to degrade microbial derived exopolysaccharides, modify their cell walls, or as a means of infiltrating plant hosts and not as method of obtaining fixed carbon. Exocellulases appear to be provided by a taxonomically narrow group of organisms including Ruminococcus, OPB54 Rhizobiales, and Actinomycetales. These bacteria have been identified as lignocellulose degrading organisms in a variety of environments and likely represent the true cellulolytic consortium of the enteric microbial community despite comprising a relatively small fraction of the overall community (<1–17%) (Ben David et al., 2015; Joshi et al., 2018; Šimůnek et al., 2018).

Carbohydrate active enzyme profiles suggest that *P. nigrolineatus* derives most of its nutrition from the hydrolysis of hemicellulose and not cellulose. At L3, the majority (9/15) of functional categories were differentially represented across the feeding diets. More abundant in the wood diet were genes involved in the metabolism of amino and nucleotide sugars, fructose, mannose, galactose, sucrose, and starches, as well as the interconversion of pentose sugars and glucuronic acid. Most of these carbohydrates have been identified as major components of xyloglucan, glucomannan, mannan, xylan, and arabinoxylan, which, together, form hemicellulose (Fry, 1989; Moreira and Filho, 2008; Rennie and Scheller, 2014). A similar pattern of gene enrichment was observed in the GI tract of giant pandas whose microbiome is replete with amylases and hemicellulases (Zhang et al., 2018). Also similar to *P. nigrolineatus*, the giant panda microbiome lacks the abundance of cellulases observed in other herbivores (Zhang et al., 2018). Coupled with a short gut transit time, it is unlikely that *P. nigrolineatus* is capable of sufficient cellulose hydrolysis. Rather, it is more likely that the limited numbers of cellulases provided by the microbiome are used to liberate the more easily hydrolysable and assimilable hemicellulose from the lignocellulose matrix.

Consumption of the higher protein mixed diet by *P. nigrolineatus* selected for a microbiome capable of amino acid catabolism and fermentation. The microbiome of mixed diet-fed fish was enriched for genes involved in the metabolism and degradation of several amino acids including branched-chain amino acids (valine, leucine, and isoleucine), tyrosine, tryptophan, lysine, histidine, glycine, serine, and threonine. Amino acid fermentation typically occurs in the distal intestines where bacterial densities are high and carbohydrate concentrations are minimal. The substrates and products of amino acid fermentation vary depending on diet, gut environment, and microbial consortium (Dai et al., 2011; Neis et al., 2015). However, the preferred amino acids include glutamine, asparagine, lysine, serine, threonine, arginine, glycine, histidine, and the branched-chain amino acids. Catabolism usually includes both deamination and decarboxylation and results in the formation of various products including ammonia, as well as short-chained/branched fatty acids and organic acids (Dai et al., 2011). Examination of the predicted metagenome (KEGG L3) showed that the mixed-diet fed fishes were enriched for genes involved in the metabolism of fatty acids, butyrate, propionate, and pyruvate. Products from the fermentation of amino acids are likely used as precursors for gluconeogenesis in mixed diet-fed fish as there is also an increased abundance of genes associated with the glyoxylate cycle.

Intestinal bacteria play a major role in host nutrition by serving as a source of essential vitamins and nutrients. Plant-based diets typically lack several compounds including sterols, B vitamins, and nitrogenous compounds, many of which cannot be synthesized by the host. Genomic and culture-based studies have identified several vitamin-producing bacteria from the GI tracts of wood-feeding invertebrates as well as herbivorous and omnivorous fish (Sugita et al., 1991; Rosenthal et al., 2011; Abhishek et al., 2015). Administering antibiotics to these wood-eating organisms often results in reduced rates of intestinal vitamin biosynthesis, strongly implicating the gut microbiome as the primary source (Lovell and Limsuwan, 1982). Dietary factors such as feed type, nitrogen content, and age have also been shown to effect vitamin synthesis in ruminant animals (Schwab et al., 2006; Beaudet et al., 2016). In this study, there was no significant increase in the relative abundance of genes associated with cofactors and vitamin metabolism in the wood-fed fish relative to the mixed-diet fish (KEGG L2). However, closer examination revealed that the mixed diet-fed fish microbiome was enriched with genes involved in ubiquinone and other terpenoid-quinone biosynthetic pathways. Included in this collection of genes are the biosynthetic pathways for α-tocopherol (vitamin E), menaquinone (vitamin K2), and phylloquinone (vitamin K1). Ubiquinone, in addition to its role in electron transport, may play a role in dissimilatory lignin degradation (DeAngelis et al., 2013).

Genes associated with xenobiotic degradation were more abundant in the mixed diet-fed fish. These findings differ from metatranscriptomic studies in termites, which showed increased expression of several detoxifying enzymes when fed bulk wood instead of less refractory foods such as paper (Raychoudhury et al., 2013). Lignin, a structural component of plant cell walls, is a major barrier to the liberation and saccharification of cellulose and hemicellulose. It is a heterogeneous, highly recalcitrant, polymer of radically coupled aromatic compounds. Degradation of lignin releases several cytotoxic and anti-nutritive compounds including organic acids, phenolic compounds, and reactive oxygen species that disrupt cellular processes and damage cell components (Abhishek et al., 2015). While relatively little is known about bacterial lignin-active enzymes, bacteria have been shown to play a major role in the degradation of lignin in several environments (Bugg et al., 2011; Abhishek et al., 2015). Of the selected lignocellulose-active enzymes examined in this study, those with activity against lignin were the most abundant, including cytochrome c peroxidases, glycolate oxidases, peroxiredoxin, and the recently described catalase-peroxidases. However, there were no significance differences in the relative abundance of these genes across diets or tissue regions, although the metagenomic analysis was limited to the bacterial fraction of the microbiome. In termite systems, the largest increases in expression of xenobiotic degrading enzymes were observed in the gut flagellate and host transcript pools (Brown and Chang, 2014). While there is no evidence of flagellates in the *P. nigrolineatus* GI tract, a diverse fungal community has been detected in all regions of the gut (Marden et al., 2017) and fungi have tremendous capacity for lignocellulose degradation, with the potential of playing an active role in lignin processing (Cragg et al., 2015).

Previous studies have intimated that wood consumption by *P. nigrolineatus* occurs simply as a means of accessing bacterial and fungal hyphae present below the wood surface and that wood consumption causes starvation in the fish (Tartar et al., 2009). In this study, fungal and photosynthetic bacterial growth was inhibited by rearing fish in the dark and autoclaved, and sterile wood was provided as the sole food source, thereby alleviating the likelihood of obtaining nutrients from wood-related microorganisms. Loricariids have a remarkable capacity to survive extended periods without food and could have easily survived the duration of the feeding experiment without consuming any food, doing so through a combination of reduced metabolic rates and reduced GI tract mucosal and microvilli surface areas (Lujan et al., 2011). Although we did not address fish dietary performance, predictive metagenomic analysis suggested that the fish did not appear to be exhibiting a “starved” phenotype. Bacterial metagenomes of starved fish have been shown to have lower abundances of genes involved in transcription, cell division, and DNA replication/repair, while genes involved in membrane transport and protein turnover are enriched (Xia et al., 2014), patterns of relative gene abundances that were not observed between the wood- and mixed-diet fed in fish. However, these measures of gene abundance are predictive and calculated from the relative abundance of different taxonomic lineages. Understanding the metabolic potential of the enteric bacterial community would benefit from a metatranscriptomic analysis of a larger number of fish.

Results demonstrated the functional resiliency of the *P. nigrolineatus* enteric bacterial community. Despite large, diet-induced, shifts in community composition, little change was observed in the predicted relative abundance of genes related to lignocellulose degradation. Our findings suggest that the enteric community composition is altered by the metabolic capacity of the microorganisms, with the GI tract environment selecting for overall community function and not specific microbial lineages. Selection based on function may serve as an advantage for wood-feeding organisms like *P. nigrolineatus* that switch between feeding habits as it insures the presence of essential metabolic pathways even after prolonged feeding on less refractory foods. How the *P. nigrolineatus* GI tract selects for lignocellulose-degrading microorganisms despite changing diets is unclear and the focus of future investigations.

## Data Availability Statement

The datasets generated during and/or analyzed during the current study are available from the NCBI Sequence Read Archive database under accession numbers PRJNA407967 (feeding study 1) and PRJNA549277 (feeding study 2).

## Ethics Statement

Fish growth conditions and all experimental protocols were approved by the Towson University Institutional Animal Care and Use Committee (IACUC 071509JW-01) and the University of Maryland School of Medicine Office of Animal Welfare Assurance (IACUC #0618005). All methods were performed in accordance with IACUC guidelines and regulations.

## Author Contributions

RM, JW, and HS conceived the project and designed research. HS and JW supervised the study. RM and HS performed research. RM, JW, and HS guided the analysis. RM, JW, and HS analyzed data. RM prepared figures. RM, JW, and HS wrote, reviewed and edited the paper. HS provided lab space. HS and JW provided funding. All authors reviewed and approved the final manuscript.

### Conflict of Interest

The authors declare that the research was conducted in the absence of any commercial or financial relationships that could be construed as a potential conflict of interest.
