# Corrigendum: Effect of Diet on the Enteric Microbiome of the Wood-Eating Catfish *Panaque nigrolineatus*

**DOI:** 10.3389/fmicb.2020.00331

**Published:** 2020-02-27

**Authors:** Ryan C. McDonald, Joy E. M. Watts, Harold J. Schreier

**Affiliations:** ^1^Department of Biological Sciences, University of Maryland, Baltimore County, Baltimore, MD, United States; ^2^Department of Biological Sciences, University of Portsmouth, Portsmouth, United Kingdom; ^3^Department of Marine Biotechnology, University of Maryland, Baltimore County, Baltimore, MD, United States

**Keywords:** lignocellulose digestion, microbiome, 16S rRNA gene amplicon sequencing, predictive metagenomics, Amazonian catfish

In the original article, there was a mistake in Table 3 as published. All exponents were incorrectly shown as positive values when they should have been negative. The corrected Table 3 appears below.

**Table 3:** Relative abundance of the three classes of cellulose degrading enzymes based on predictive metagenomics.

	**Mixed diet**	**Wood diet**
Endoglucanase (EC:3.2.1.4)	4.01 × 10^−4^ ± 1.43 × 10^−4^	4.50 × 10^−4^ ± 8.74 × 10^−5^
β-Glucosidase (EC:3.2.1.21)	1.25 × 10^−4^ ± 9.21 × 10^−5^	2.59 × 10^−4^ ± 1.90 × 10^−4^
Cellulose 1,4-β-cellobiosidase (EC:3.2.1.91)	1.04 × 10^−7^ ± 2.03 × 10^−7^	6.19 × 10^−7^ ± 8.98 × 10^−7^

*Abundances were calculated using PICRUSt (see methods) and compared across diet type and tissue region*.

The authors apologize for this error and state that this does not change the scientific conclusions of the article in any way. The original article has been updated.

