# Supplementary material for: Effect of Diet on the Enteric Microbiome of the Wood-Eating Catfish Panaque nigrolineatus
Source: Front Microbiol. 2019 Nov 29;10:2687. doi: 10.3389/fmicb.2019.02687 (PMC6895002; doi:10.3389/fmicb.2019.02687)
Supplement: Supplementary file 1 [file Data_Sheet_1.zip › Data_Sheet_1/Data Sheet 1/Supplemantary_Figure_6_updated.docx]

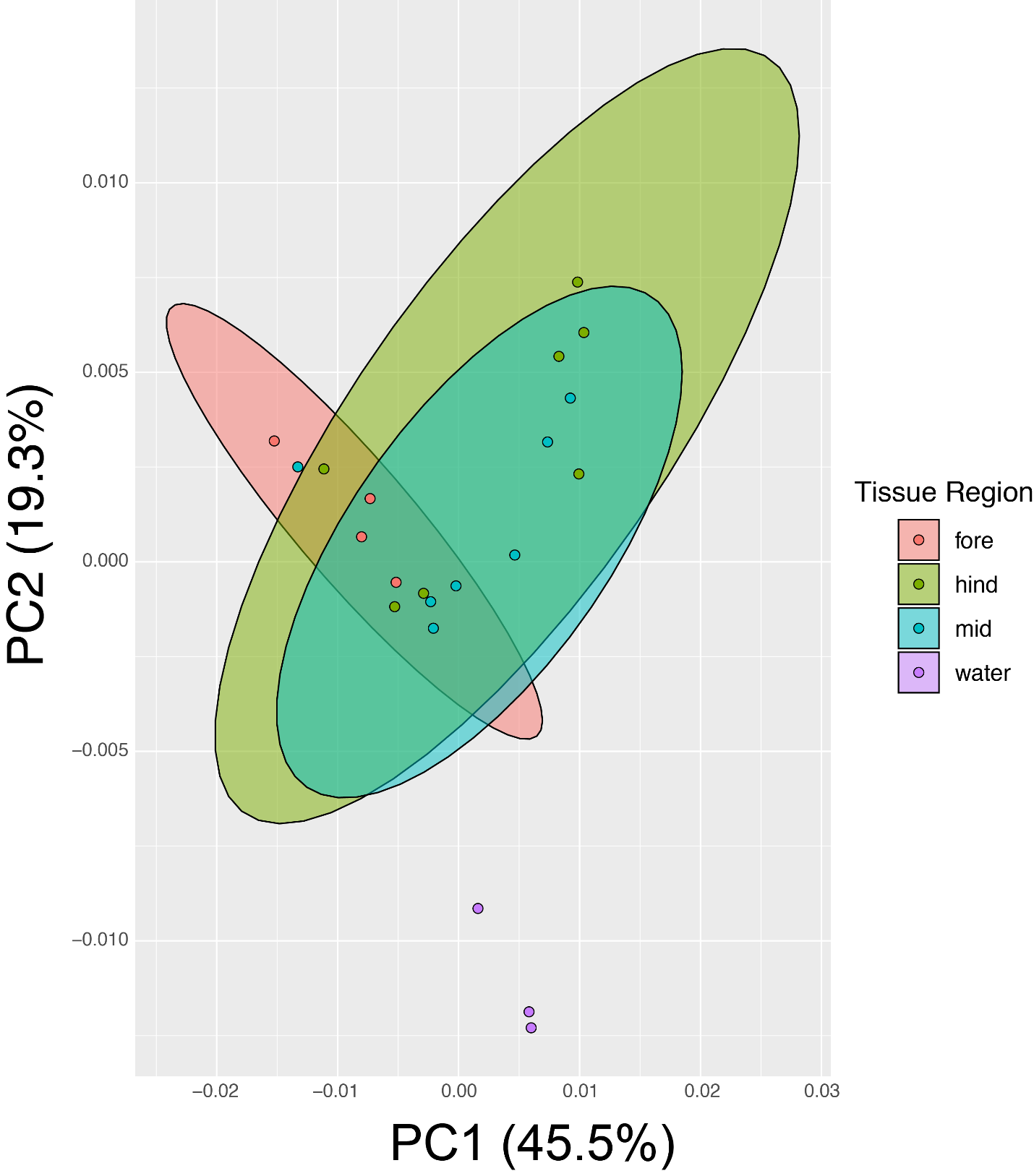


**Supplemantary Figure 6.** Principal component analysis (PCA) biplot of PICRUSt predicted metagenomes of wood and mixed diet-fed fish. A 0.95 confidence grouping variable ellipses was drawn for the foregut (fore), midgut (mid), hindgut (hind), and tank water (water). With the exception of tank water, predicted metagenomes did not cluster based tissue region for PC1 and PC2.
