# Supplementary material for: Effect of Diet on the Enteric Microbiome of the Wood-Eating Catfish Panaque nigrolineatus
Source: Front Microbiol. 2019 Nov 29;10:2687. doi: 10.3389/fmicb.2019.02687 (PMC6895002; doi:10.3389/fmicb.2019.02687)
Supplement: Supplementary file 1 [file Data_Sheet_1.zip › Data_Sheet_1/Data Sheet 1/Supplementary_Figure_2_update.docx]

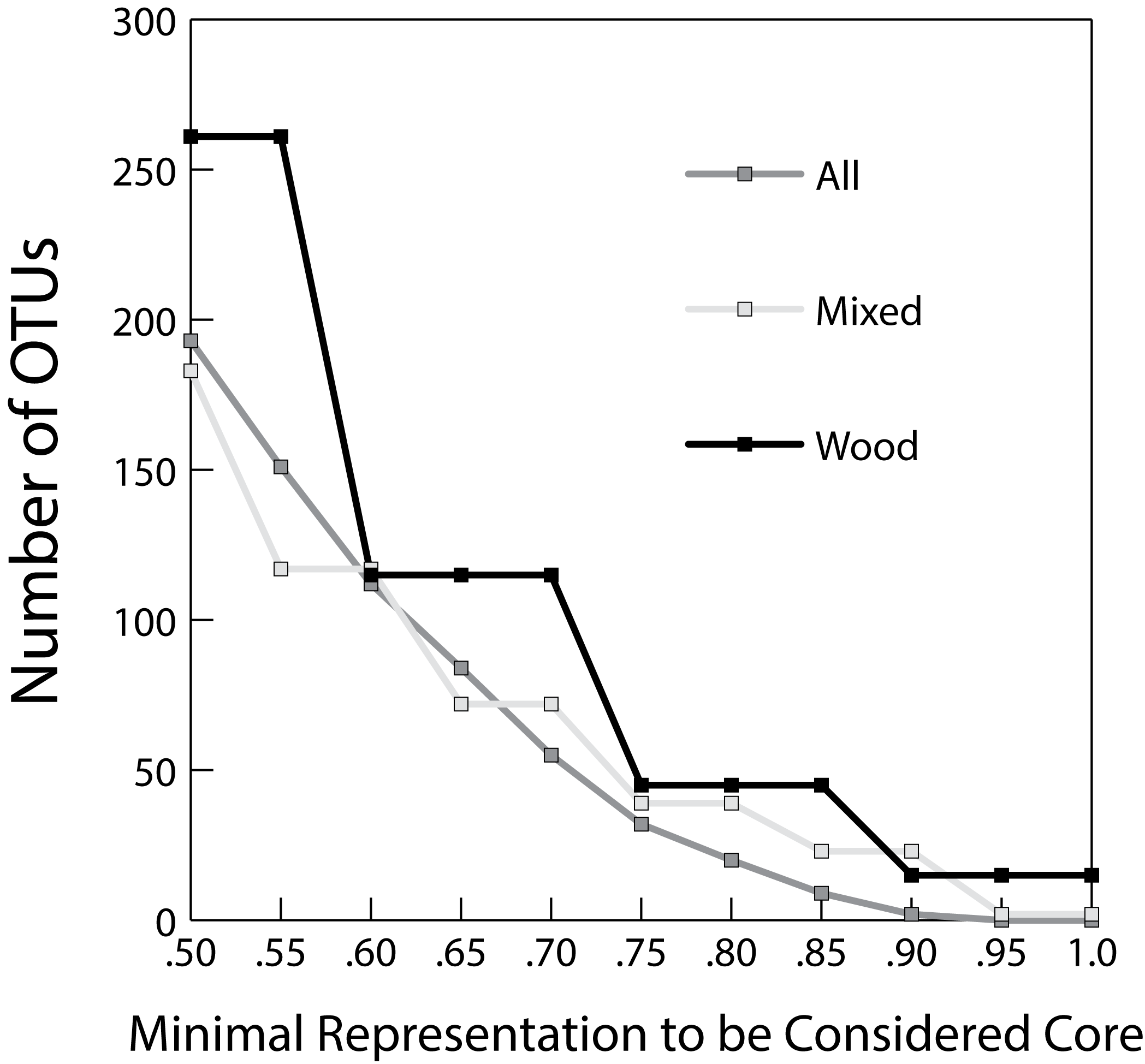


**Supplementary Figure 2.** ***P. nigrolineatus* fed a wood diet possess an expanded core microbiome as indicated by the increased number of OTUs shared amongst the majority of samples.**
