# Supplementary material for: Effect of Diet on the Enteric Microbiome of the Wood-Eating Catfish Panaque nigrolineatus
Source: Front Microbiol. 2019 Nov 29;10:2687. doi: 10.3389/fmicb.2019.02687 (PMC6895002; doi:10.3389/fmicb.2019.02687)
Supplement: Supplementary file 1 [file Data_Sheet_1.zip › Data_Sheet_1/Data Sheet 1/Supplementary_Figure_3_update.docx]

**Supplementary Figure 3.** **Venn diagram depicting the shared OTUs identified in the core microbiomes of wood and mixed diet-fed fish. OTUs must be present in a at least 70% of samples to be considered a member of the core microbome.**
