# Supplementary material for: Effect of Diet on the Enteric Microbiome of the Wood-Eating Catfish Panaque nigrolineatus
Source: Front Microbiol. 2019 Nov 29;10:2687. doi: 10.3389/fmicb.2019.02687 (PMC6895002; doi:10.3389/fmicb.2019.02687)
Supplement: Supplementary file 1 [file Data_Sheet_1.zip › Data_Sheet_1/Data Sheet 1/Supplementary_Figure_4_update.docx]

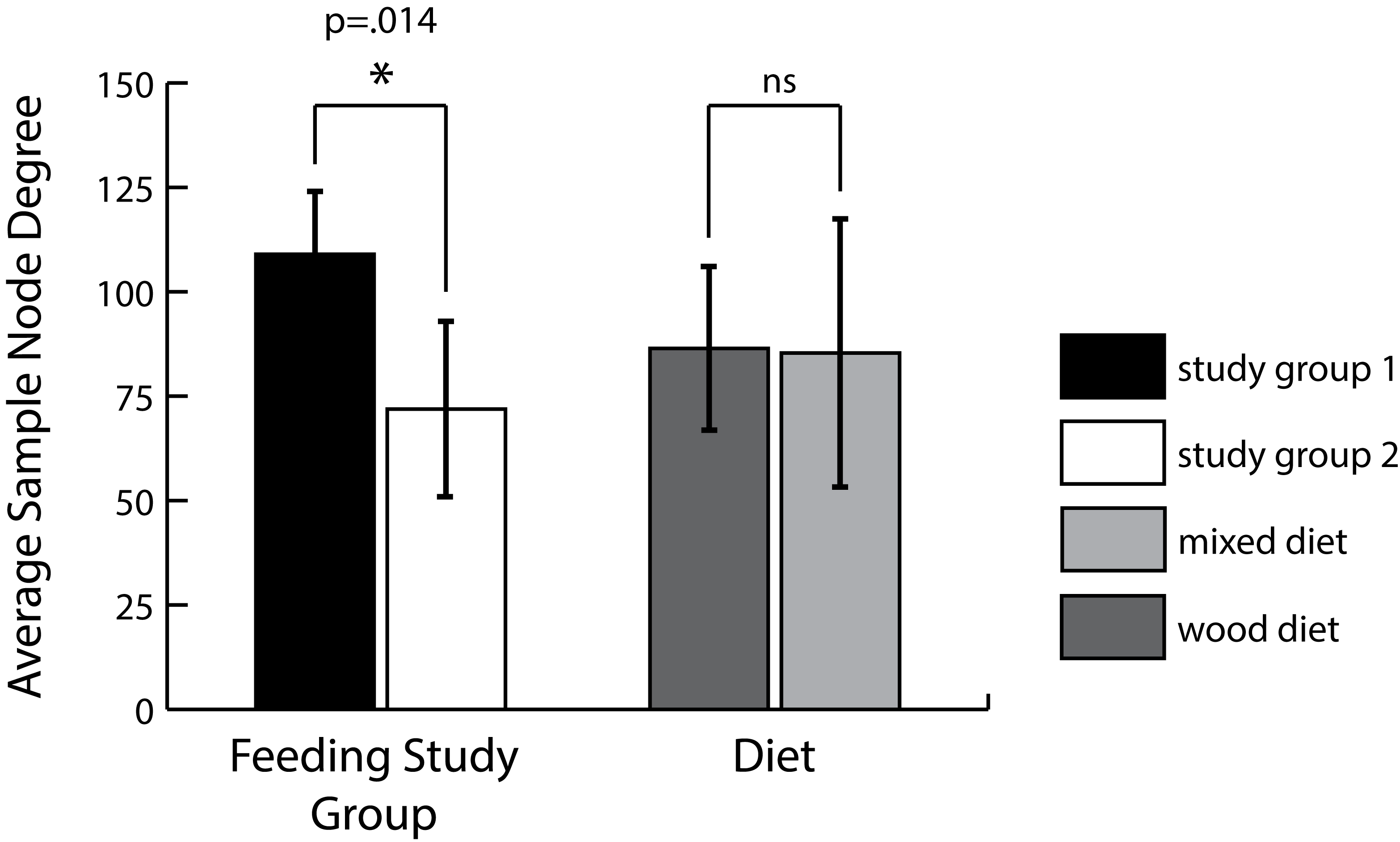


**Supplementary Figure 4.** **Enteric microbial diversity was significantly reduced in second feeding study as indicated by reduced average sample node degree distribution. No significant difference in sample node degree distribution was observed between diets. These values were calculated from an OTU network constructed using a rarefied BIOM table suggesting differences in diversity are not a result of differences in sampling depth.**
