# Supplementary material for: Effect of Diet on the Enteric Microbiome of the Wood-Eating Catfish Panaque nigrolineatus
Source: Front Microbiol. 2019 Nov 29;10:2687. doi: 10.3389/fmicb.2019.02687 (PMC6895002; doi:10.3389/fmicb.2019.02687)
Supplement: Supplementary file 1 [file Data_Sheet_1.zip › Data_Sheet_1/Data Sheet 1/Supplementary_table_5.docx]

| KEGG Hierarchy (L2) | KEGG Hierarchy (L3) | Mixed Diet Average Relative Abundance | Wood Diet Average Relative Abundance | p-value |
| --- | --- | --- | --- | --- |
| Membrane Transport | Secretion system | 1.8x10^2^ ± 5.1x10^3^ | 2.4x10^2^ ± 3.1x10^3^ | 0.03 |
| Sorting and Degradation | Chaperones and folding catalysts | 8.7x10^3^ ± 1.1x10^3^ | 9.8x10^3^ ± 4.4x10^4^ | 0.03 |
|  | Ubiquitin system | 2.0x10^4^ ± 6.4x10^5^ | 2.7x10^4^ ± 2.2x10^5^ | 0.02 |
| Replication and Repair | Non-homologous end-joining | 5.6x10^4^ ± 2.2x10^4^ | 3.2x10^4^ ± 1.6x10^4^ | 0.04 |
| Amino Acid Metabolism | Glycine, serine and threonine metabolism | 9.7x10^3^ ± 3.8x10^4^ | 9.1x10^3^ ± 4.5x10^4^ | 0.03 |
|  | Histidine metabolism | 5.3x10^3^ ± 4.3x10^4^ | 4.6x10^3^ ± 5.6x10^4^ | 0.03 |
|  | Lysine degradation | 6.0x10^3^ ± 1.9x10^3^ | 4.1x10^3^ ± 5.9x10^4^ | 0.02 |
|  | Tryptophan metabolism | 7.1x10^3^ ± 2.00x10^3^ | 5.2x10^3^ ± 5.7x10^4^ | 0.03 |
|  | Tyrosine metabolism | 5.1x10^3^ ± 5.0x10^4^ | 4.4x10^3^ ± 1.7x10^4^ | <0.01 |
|  | Valine, leucine and isoleucine degradation | 9.94x10^3^ ± 2.8x10^3^ | 6.2x10^3^ ± 1.0x10^3^ | <0.01 |
| Biosynthesis of Other Secondary Metabolites | Isoquinoline alkaloid biosynthesis | 6.9x10^4^ ± 6.2x10^5^ | 5.8x10^4^ ± 3.7x10^5^ | <0.01 |
|  | Tropane, piperidine and pyridine alkaloid biosynthesis | 1.3x10^3^ ± 2.2x10^4^ | 1.1x10^3^ ± 7.9x10^5^ | 0.02 |
| Carbohydrate Metabolism | Amino sugar and nucleotide sugar metabolism | 9.0x10^3^ ± 7.9x10^4^ | 1.0x10^2^ ± 4.7x10^4^ | <0.01 |
|  | Butanoate metabolism | 1.2x10^2^ ± 2.4x10^3^ | 9.0x10^3^ ± 1.4x10^3^ | 0.04 |
|  | Fructose and mannose metabolism | 6.0x10^3^ ± 6.9x10^4^ | 7.2x10^3^ ± 9.8x10^4^ | 0.03 |
|  | Galactose metabolism | 3.1x10^3^ ± 2.8x10^4^ | 4.0x10^3^ ± 6.3x10^4^ | 0.02 |
|  | Glyoxylate and dicarboxylate metabolism | 7.8x10^3^ ± 8.7x10^4^ | 6.7x10^3^ ± 2.7x10^4^ | <0.01 |
|  | Pentose and glucuronate interconversions | 4.2x10^3^ ± 3.8x10^4^ | 5.2x10^3^ ± 7.2x10^4^ | 0.02 |
|  | Propanoate metabolism | 1.1x10^2^ ± 2.4x10^3^ | 8.0x10^3^ ± 8.1x10^4^ | 0.02 |
|  | Pyruvate metabolism | 1.12x10^2^ ± 9.1x10^4^ | 1.1x10^2^ ± 3.0x10^4^ | 0.02 |
|  | Starch and sucrose metabolism | 5.3x10^3^ ± 7.4x10^4^ | 6.4x10^3^ ± 8.3x10^4^ | 0.03 |
| Energy Metabolism | Carbon fixation pathways in prokaryotes | 1.1x10^2^ ± 1.0x10^3^ | 9.6x10^3^ ± 4.6x10^4^ | 0.02 |
| Glycan Biosynthesis and Metabolism | Other glycan degradation | 5.0x10^4^ ± 1.7x10^4^ | 7.2x10^4^ ± 9.4x10^5^ | <0.01 |
| Lipid Metabolism | Ether lipid metabolism | 1.1x10^4^ ± 6.8x10^5^ | 2.8x10^5^ ± 2.0x10^5^ | 0.01 |
|  | Fatty acid metabolism | 9.0x10^3^ ± 3.2x10^3^ | 6.1x10^3^ ± 1.0x10^3^ | 0.04 |
|  | Linoleic acid metabolism | 9.8x10^4^ ± 3.3x10^4^ | 5.6x10^4^ ± 2.6x10^4^ | 0.02 |
|  | Synthesis and degradation of ketone bodies | 1.8x10^3^ ± 5.5x10^4^ | 8.7x10^4^ ± 2.4x10^4^ | <0.01 |
| Metabolism of Cofactors and Vitamins | Porphyrin and chlorophyll metabolism | 9.5x10^3^ ± 6.2x10^4^ | 8.2x10^3^ ± 4.1x10^4^ | <0.01 |
|  | Ubiquinone and other terpenoid-quinone biosynthesis | 3.4x10^3^ ± 3.6x10^4^ | 4.2x10^3^ ± 3.9x10^4^ | <0.01 |
| Xenobiotics Biodegradation and Metabolism | Chloroalkane and chloroalkene degradation | 3.9x10^3^ ± 8.4x10^4^ | 2.8x10^3^ ± 7.3x10^4^ | 0.02 |
|  | Drug metabolism - cytochrome P450 | 2.2x10^3^ ± 4.7x10^4^ | 1.6x10^3^ ± 1.7x10^4^ | 0.01 |
|  | Metabolism of xenobiotics by cytochrome P450 | 2.0x10^3^ ± 4.2x10^4^ | 1.6x10^3^ ± 1.8x10^4^ | 0.02 |
|  | Styrene degradation | 1.1x10^3^ ± 2.7x10^4^ | 6.9x10^4^ ± 2.2x10^4^ | 0.01 |
| Cellular Processes and Signaling | Inorganic ion transport and metabolism | 4.2x10^3^ ± 6.7x10^4^ | 5.0 x10^3^ ± 3.0x10^4^ | 0.02 |
|  | Signal transduction mechanisms | 4.9x10^3­­^ ± 4.7x10^4^ | 5.7x10^3^ ± 6.9x10^4^ | 0.04 |

**Supplementary Table 5.** Predictive functional profiles were generated from 16S rRNA marker gene sequences using PICRUSt. Statistically significant differences (two-tailed Student’s t-test; p < .05) were detected between wood and mixed diet-fed fish at L3 within the KEGG hierarchy.
